# Manufacturing of peptides exhibiting biological activity

**DOI:** 10.1007/s00726-012-1379-7

**Published:** 2012-08-23

**Authors:** Aleksandra Zambrowicz, Monika Timmer, Antoni Polanowski, Gert Lubec, Tadeusz Trziszka

**Affiliations:** 1 Department of Animal Products Technology and Quality Management, Wroclaw University of Environmental and Life Sciences, Chelmonskiego 37/41, 51-630 Wroclaw, Poland; 2 Department of Pediatrics, Medical University of Vienna, Waehringer Guertel 18, 1090 Vienna, Austria

**Keywords:** Bioactive peptides, Enzymatic hydrolysis, Immunostimulatory activity, Ovokinin

## Abstract

Numerous studies have shown that food proteins may be a source of bioactive peptides. Those peptides are encrypted in the protein sequence. They stay inactive within the parental protein until release by proteolytic enzymes (Mine and Kovacs-Nolan in Worlds Poult Sci J 62(1):87–95, 2006; Hartman and Miesel in Curr Opin Biotechnol 18:163–169, 2007). Once released the bioactive peptides exhibit several biofunctionalities and may serve therapeutic roles in body systems. Opioid peptides, peptides lowering high blood pressure, inhibiting platelet aggregation as well as being carriers of metal ions and peptides with immunostimulatory, antimicrobial and antioxidant activities have been described (Hartman and Miesel in Curr Opin Biotechnol 18:163–169, 2007). The biofunctional abilities of the peptides have therefore aroused a lot of scientific, technological and consumer interest with respect to the role of dietary proteins in controlling and influencing health (Möller et al. in Eur J Nutr 47(4):171–182, 2008). Biopeptides may find wide application in food production, the cosmetics industry as well as in the prevention and treatment of various medical conditions. They are manufactured by chemical and biotechnological methods (Marx in Chem Eng News 83(11):17–24. 2005; Hancock and Sahl in Nat Biotechnol 24(12):1551–1557, 2006). Depending on specific needs (food or pharmaceutical industry) different degrees of peptide purifications are required. This paper discusses the practicability of manufacturing bioactive peptides, especially from food proteins.

## Food protein enzymatic hydrolysis

Recently, there has been a significant increase of studies on enzymatic hydrolysis (in vitro) to release bioactive peptides from food proteins (Spellman et al. 2003). Bioactive peptides are released from proteins during some food processing such as cheese ripening or milk fermentation (Hartman and Miesel 2007). The use of enzymatic hydrolysis is preferred, especially in food and pharmaceutical industries, because of the lack of residual organic solvents and toxic chemicals in the final peptide preparations (Agyei and Danquah 2011).

The consequence of the proteolytic action is the change in molecular conformation of native proteins and production of functional and bioactive products (Panyam and Kilara 1996; Adebiyi et al. 2008). Enzymatic hydrolysis is a process conducted under mild conditions which can be easily controlled and allows one to obtain products with well-defined features (Clemente 2000; FitzGerald and O’Cuinn 2006). Most enzymatic modifications of dietary proteins are carried out by enzymes such as pepsin, bromelain, trypsin, chymotrypsin, papain or ficain under their respective optimal pH and temperature conditions (Chrzanowska 1998). However, the application of enzymes can increase the cost of the process, which is why cheap sources of them are preferred. One of the best sources of animal origin proteinases are pancreases, by-products of the meat industry (Żelazko et al. 2007). Also microorganisms are a relatively cheap source of proteases. For example, Neutrase, Subtilisin, Orientase, Protex 7L, Protamex 1.5 and proteases from lactic acid bacteria (LAB) are widely used for the production of bioactive peptides (Chrzanowska 1998; Sakanaka and Tachibana 2006; Wagar et al. 2009). Microbial proteases provide few advantages over proteases from other sources. Bacterial cultivation costs are relatively low (minimal nutritional requirements, short time of maturation) and proteases, especially of the LAB, are expressed on the cell membrane making purification protocols relatively easy and cheap. Furthermore LAB and their products are considered safe (generally recognized as safe, GRAS) (Agyei and Danquah 2011).

The precursors of biopeptides may be proteins of animal origin such as milk proteins (casein, whey protein), muscle proteins (myosin, collagen) as well as of plant origin such as soy protein (glycinin, β-conglycinin), wheat and rice protein (Gibbs et al. 2004; Ibrahim et al. 1998; Kim et al. 2007; Kong et al. 2007; Li et al. 2007; Saiga et al. 2003). Because of their potential as physiological modulators, as well as the high safety profile, they can be used as components of functional foods and nutraceuticals. Bioactive peptides can be also released from protein by-products of food industry, reducing the production costs with the added advantage of an efficient waste disposal. Peptic hydrolysis of rapeseed protein (oil industry waste) leads to the generation of peptides possessing strong ACE inhibitory and DPPH radical scavenging activity (Yoshie-Stark et al. 2006). Hydrolysates produced with Orientase and protease from *Bacillus* sp. of delipidated egg yolk protein, as obtained by lecithin extraction, exhibited DPPH and hydroxyl radical scavenging activity (Sakanaka and Tachibana 2006).

The in vitro enzymatic hydrolysis and the in situ microbial fermentation of food proteins both give rise to mixtures of peptides. However, the complexity of the starting hydrolysate makes the purification process quite demanding and time-consuming; in numerous situations each biopeptide might require a complicated process protocol (Agyei and Danquah 2011). Currently, the standard protocol for bioactive peptide isolation includes precipitation of unhydrolysed protein (Graszkiewicz et al. 2007), membrane filtration (ultrafiltration) (Xu et al. 2007), liquid chromatography (including ion-exchange, gel filtration chromatography (Xu et al. 2007; Amarowicz and Shahidi 1997) and in particular, reversed-phase high-performance liquid chromatography (RP-HPLC) (Adebiyi et al. 2008). Purification protocols for antioxidant peptides from the papain hydrolysate of pork muscles described by Saiga et al. (2003) involves cation (AG 50 W-X2 resin) with subsequent anion exchange chromatography (AG 1-X4 resin), and the resulting fractions are further applied to an ultrafiltration membrane (MW < 500 Da) and finally separated on an RP-HPLC column. However, Adebiyi et al. (2008) fractionated the rice bran hydrolysates to pure antioxidative peptides by RP-HPLC on a Kaseigel ODS resin, using a stepwise gradient of aqueous ethanol only. This approach usually results in good results in laboratory scale purification; however, sometimes it is not easy to translate it into large scale production because of the poor yields. On the other hand, the lower production costs as compared to chemical synthesis of the biopeptides make those techniques worthy of consideration (Agyei and Danquah 2011).

In many cases in the food industry, whole hydrolysates (unpurified bioactive peptides) are the main components of functional food. Fermented milks such as Calpis AMEEL S (Japan) and Evolus (Finland) or the energy drink CholesteBlock (Japan), which exert antihypertensive and antihyperholesterolemic effects, respectively, are available on the market. Pure peptides found application only in pharmacy. A representational example is the LKPN peptide named “Katshuobushi Oligopeptide” released upon Thermolysin treatment of Bonito fish. It is an active component of the antihypertensive drugs Vasotensin 120T™, PeptACE™ and Peptides 90 (Hartman and Miesel 2007).

There are many advantages of natural peptides. They are mainly toxicologically safe, have a wide spectra of therapeutic action, exhibit less side effects as compared to synthetic drugs and are better absorbed in the intestinal tract (Erdman et al. 2008; Agyei and Danquah 2011). For example, ovokinin, a peptide isolated from the peptic hydrolysate of egg white, reduces blood pressure, without side effects or induction of hypotension. Therefore, it may be administered to patients with moderate hypertension or with regular blood pressure (Erdman et al. 2008). The synthetic peptides have a prolonged retention time out of body tissues and may also interact with other medications used by patients (Erdman et al. 2008; Agyei and Danquah 2011).

The unavailability of large scale technologies and high cost of purification techniques are limiting factors to the commercialization of food-derived bioactive peptides. Research on the isolation of bioactive peptides reducing time and costs are continuously developing (Agyei and Danquah 2011). One possible solution is the application of immobilized enzymes. Proteolysis on the resin enables recycling of the enzyme and at the same time generation of secondary metabolites originating from autolysis of enzymes is avoided (Agyei and Danquah 2011). Immobilized enzymes have been effectively used to produce bioactive peptides from *Brassica carinata* proteins (Pedroche et al. 2007).

Another common approach is the use of continuous processes rather than conventional batch methods to hydrolyse the proteins. Kapel et al. (2006) produced antihypertensive and opioid peptides from Alfalfa (*Medicago* *sativa*) white protein concentrate. Hydrolysis was carried out in a reactor coupled to two tubular ultrafiltration modules. Cui et al. (2011) effectively performed continuous hydrolysis to obtain bioactive peptides from wheat gluten. The permeate fractions showed antioxidant activities that were mostly due to the low molecular weight peptides. The enzyme membrane reactor system offers control of the reaction with minimized substrate and catalyst losses, faster reactions and higher yields, cleaner products and lower operating costs (Rios et al. 2004).

## Food-derived biologically active peptides

Numerous food-derived peptides exhibiting activities such as opiate, antithrombotic, anticancer, antihypertensive, immunomodulation, mineral-binding, antimicrobial or antioxidant properties have been reported (Hartman and Miesel 2007).

Research has focused on novel peptides with pharmacological properties. The group of antithrombotic peptides derived from bovine κ-casein (106–116; 106–112; 112–166 and 113–116) are named casoplatelins. One of them, Casopiastrin (ft106–116) released from κ-casein during the proteolytic action of trypsin, exerts antithrombotic activity by inhibition of the fibrinogen binding process whereas another peptide (103–111) of this protein inhibited platelet aggregation (Iwaniak and Minkiewicz 2007).

Short peptides with cationic and hydrophobic properties are recognized as potential antibiotics. Peptides with antimicrobial and antiviral activity have been reported for certain food proteins. Lactoferricin B, comprising residues from 17 to 41 in the sequence of milk lactoferrin, has been found to exert antimicrobial activity. As a result of aggregation of peptides in the cytoplasmic membrane, channels are formed which destabilize bacterial membranes thus leading to microbial cell death (Chrzanowska 1998). The same effect is observed for the 92-amino acid ovotransferrin peptide, OTAP-92, which is capable of killing gram-negative bacteria. It also shows antiviral activity against Marek’s disease virus in chicken embryo fibroblasts (Mine and Kovacs-Nolan 2006). A good example of an antimicrobial peptide is a cysteine-rich peptide obtained by hydrolysis of oysters with alcalase and bromelain which inhibits the growth of both gram-positive and gram-negative bacteria (Liu et al. 2008). Kobayashi et al. (2004) isolated a highly glycosylated peptides from ovomucin by pronase treatment and reported that peptide exhibited antiadhesive and antiviral activity towards bovine rotavirus, Newcastle and influenza viruses.

Milk proteins are a rich source of opioid peptides. Those peptides are released from casein and whey protein. The largest group are β-casomorphins released from β-casein. They show the same amino terminal sequence (Tyr-Pro-Pro-Fen); their bioactivity is determined by the presence of tyrosine residues in the N-terminal position. Opioid peptides act on the central nervous system, affect pain perception and behaviour. These peptides also act locally in the gastrointestinal system, e.g. they extend the transit time of ingesta through the digestive tract (Chrzanowska 1998).

A number of studies have been performed on the immunomodulatory activity of peptides (Quian et al. 2011; Wagar et al. 2009). Many of them significantly enhance lymphocyte proliferation, natural killer cell activity, and the secretion of cell cytokines. Peptides derived from hen egg ovomucoid hydrolysate induced T cell formation. Also immunomodulating peptides were obtained from peptic (CN77-84) and chymotryptic (CN126-134) hydrolysates of ovalbumin (Mine and Kovacs-Nolan 2006).

Human digestive tract cancers are strongly influenced by diet (Rose et al. 2007). Several peptides with anticancerogenic activity have been found in food protein hydrolysates (Yang et al. 2008; Agyei and Danquah 2011). Egg yolk proteins and their hydrolysates exhibit antitumour activity by inhibiting tumour cell proliferation in the colon (Ishikawa et al. 2009). Also hydrophobic peptides extracted from soy sauce exert cytotoxic activity on human colon carcinomas and mouse lymphoma cell lines (Yang et al. 2008).

Antidiabetic peptides have been found in egg white hydrolysates obtained from alcalase digestion. The peptide RVPSLM exhibited α-glucosidase inhibitory activity with an IC_50_ value of 23.07 μmol/L (Yu et al. 2011).

Many peptides are multifunctional and exhibit more than one activity. Peptides separated from beef sarcoplasmic protein enzymatic hydrolysate show ACE inhibitory, antimicrobial and cancer cell cytotoxic effects (Yang et al. 2008). Other examples are oligophosphopeptides with molecular masses of 1–3 kDa derived from tryptic hydrolysis of phosvitin. These peptides enhanced calcium binding capacity and inhibited the formation of insoluble calcium phosphates. Oligophosphopeptides also increased iron uptake and showed novel antioxidant activity against oxidative stress in human intestinal epithelial cells in an in vitro assay using Caco-2 cells (Xu et al. 2007). All the examples given above provide probable therapeutic roles of bioactive peptides obtainable from food proteins. Proteins and precursors of food-derived peptides are well-tolerated by the human body and therefore their use in drug development may reduce costs and duration of toxicological studies during R & D and clinical trials (Agyei and Danquah 2011).

## Chemical synthesis of peptides

The past few decades have seen extraordinary progress in the chemical synthesis of peptides because of an explosive growth in biological research related to these molecules. Chemical synthesis allows the systematic variation of structure with the aim of developing peptides for therapeutic use (Kent 1988). Chemical synthesis of peptides is widely used in structural biology, immunology, protein engineering and biomedical research (Miranda and Alewood 1998). The traditional solution chemical synthesis is a time-consuming process and needs many toxic reagents, which may contribute to significant environmental pollution. Furthermore, it provides poor yields, racemization reactions and peptide by-products (Guzmán et al. 2007; Shwan 2008). The synthesis of polypeptides on a solid support is an alternative to traditional methods and dramatically improves yields, decreases the amount of chemicals used and considerably improves the fidelity of peptide chain assembly (Miranda and Alewood 1998; Guzmán et al. 2007).

Advances in solid-phase peptide synthesis (SPPS) over the past decade have encouraged increased efforts towards the total chemical synthesis of large complex peptides and small proteins. It leads to superior acylation rates, reduces racemization and has higher solubility in the common coupling solvents, e.g. DMF (Guzmán et al. 2007). Miranda and Alewood (1998) established highly efficient chemical procedures (SPPS) that achieve chain assembly rates of approximately 10–15 amino acid residues per hour, thus underpinning the rapid chemical synthesis of long polypeptides. They synthesized several small to medium peptides, including the “difficult” C-terminal sequence of HIV-1 proteinase (residues 81–99); fragment 65–74 of the acyl carrier protein; conotoxin PnIA(A10L), a potent nicotinic receptor antagonist; and the pro-inflammatory chemotactic protein CP10, an 88-residue protein, by means of native chemical ligation.

Sequence analysis of bioactive peptides can be used to predict the potential bioactivity. New peptides with improved biological activity as compared to their natural analogues can be designed. Chen et al. (1998) obtained 22 synthetic histidine-containing peptides with antioxidant properties, which were designed on the basis of the peptide (Leu-Leu-Pro-His-His) derived from proteolytic digests of a soybean protein. Another opportunity offered by chemical synthesis is substitution of particular amino acids in the peptide sequence, which can result in modulation or differentiation of peptide function. Replacement of the C-terminal phenylalanine by tryptophan in ovokinin (2–7), one of the best characterized bioactive peptides of egg, resulted in significant improvement of its antihypertensive activity (Matoba et al. 2001; Yamada et al. 2002). Replacement of the basic amino acids in particular positions of peptides increases their anticoagulant activity (Atanassov and Tchorbanov 2009). The synthesis of analogues of the antithrombotic antistasin, ghilantens and TAP by studying the role of basic and d-amino acids at different positions in the molecule for the anticoagulant activity has been described in a series of publications (Atanassov and Tchorbanov 2009).

The development of chemical synthesis leads to the availability of novel multifunctional biopeptides. Gurevich et al. (1997) developed a synthetic peptide containing both the antiadhesive Arg-Gly-Asp (RGD) amino acid sequence and a nitric oxide (NO) moiety with vasorelaxant properties. The RGD-NO peptide increased the antithrombotic characteristics of the RGD peptide. This peptide also caused relaxation of rat aortic rings (Gurevich et al. 1997).

Most therapeutic peptides manufactured by chemical synthesis have two main applications as peptide drugs and as peptides for diagnostic purposes. Currently many natural peptide analogues like the antihypertensive RPFHP and RPLKPW are available on the market (Gołąb and Warwas, 2005; Erdman et al. 2008).

## Molecular biology methods

Solid-phase peptide synthesis works quicker than classical synthesis, although still much more slowly than a living cell (Kent 1988). Currently, many studies are focusing on the development of synthetic genes for the delivery and expression of bioactive peptides or their precursors in microbial cells. This could be a good alternative to food protein hydrolysis because the amount of bioactive peptides available in food products is limited by protein concentration. For example antimicrobial lactoferricin is released during the enzymatic hydrolysis of milk lactoferrin; however, the concentration of lactoferrin is low in bovine milk (Kim et al. 2006; Renye and Somkuti 2007).

The manufacture of larger amounts of peptides needed for food supplementation may be possible by using food-grade dairy fermentation bacteria as peptide production systems. For example two peptides, the 11-residue antimicrobial peptide from bovine lactoferrin (BL-11) (RRWQWRMKKLG) and the 12-residue ACE inhibitory peptide from αs1-casein (C-12) (FFVAPFPEVFGK), have been cloned in *Streptococcus thermophilus* bacteria. Nucleic acid sequences encoding the peptides were generated by overlapping PCR and were subsequently cloned into a new expression vector (Renye and Somkuti 2007).

Lactoferricin, an antimicrobial peptide, was successfully multimerized and expressed in *Escherichia coli* BL21(DE3). About 60 mg of pure peptide with a molecular weight similar to that of the chemically synthesized lactoferricin was obtained from 1 L of *E. coli* culture (Kim et al. 2006). The successful expression of the antimicrobial peptide buforin II as a concatemeric multimer in *E. coli* was also reported (Kim et al. 2006).

Another technique for manufacturing bioactive peptides includes the expression of recombinant protein in the microbial cell followed by the hydrolysis of the fusion protein by microbial proteinase. For example recombinant lactoferricin B was cleaved successfully to a novel hybrid antimicrobial peptide LFT33 by enterokinase (Feng et al. 2011).

Bioactive peptides can be obtained directly from living organisms by the hydrolysis of proteins or by chemical synthesis and these approaches are not cost-effective. A biological expression system using genetic techniques based on a fusion technology would be a more efficient method for the production of bioactive peptides (Kim et al. 2006; Renye and Somkuti 2007). However, the molecular biology research has led to the availability of amino acid sequence data determined by the cloning and cDNA sequencing of genes. However, in many cases, the proteins themselves have never been isolated and their properties are largely unknown (Kent 1988).
